# A single coronary artery with left circumflex artery crossing right ventricular outflow tract in tetralogy of Fallot with absent left pulmonary artery

**DOI:** 10.34172/jcvtr.2020.61

**Published:** 2020-12-23

**Authors:** Vivek Jaswal, Shyam Kumar Singh Thingnam, Vikas Kumar, Ruchit Patel, Ganesh Kumar Munirathinam, Dheemta Toshkhani

**Affiliations:** ^1^Department of Cardiovascular and Thoracic Surgery, Post Graduate Institute of Medical Education and Research (PGIMER), Chandigarh, India; ^2^Department of Anaesthesia and Critical Care, Post Graduate Institute of Medical Education and Research (PGIMER), Chandigarh, India

**Keywords:** Single Coronary Artery, Left Circumflex Artery, Right Ventricular Outflow Tract, Tetralogy of Fallot, Absent Left Pulmonary Artery

## Abstract

Tetralogy of Fallot (TOF) with unilateral absence of pulmonary artery and the anomalous coronary artery is a rare combination. Detailed preoperative evaluation of coronary artery anatomy is must to prevent injury to the major vessels crossing right ventricular outflow tract. We report a rare association of single coronary artery with left circumflex artery crossing right ventricular outflow tract close to the pulmonary annulus in tetralogy of Fallot with absent left pulmonary artery in 11-year-old girl. Though there is a great diversity of coronary anomalies in tetralogy of Fallot, the prepulmonic course of left circumflex artery crossing the right ventricular outflow tract (RVOT) close to the pulmonary annulus has rarely been described in the literature. The patient underwent successful primary single lung intracardiac repair. Right ventricular outflow tract obstruction was treated by handmade valved pericardial autologous conduit and release of the tethering of hypoplastic native unicuspid pulmonary valve leaflet maintaining its integrity.

## Introduction

The incidence of anomalous coronary artery (ACA) in TOF is 2–9%.^1^ A singlecoronary artery arising from either the right or left coronary sinus is seen in 1.8-4.2% of these patients.^1^ About 2-10% of patients with TOF have an anomalous coronary artery crossing the RVOT thereby challenging the surgical skills of surgeon during RVOT reconstruction.^1,2^ TOF with unilateral absence of pulmonary artery is a rare variant with incidence of 0.95- 3.23%.^3^ No association with single coronary artery and left circumflex artery crossing the RVOT has been described in these patients.

## Case Presentation

**Figure 1:**
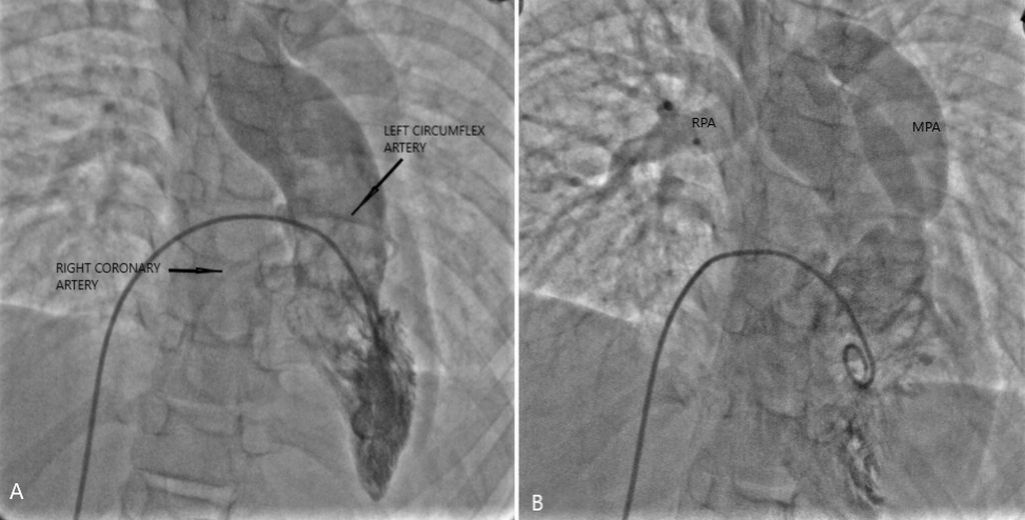
**(A)** Left ventricle angiogram showing a single coronary artery arising from right aortic sinus with anomalous left circumflex artery crossing right ventricular outflow tract **(B)** Right ventricle angiogram showing main pulmonary artery continuing as right pulmonary artery with absent left pulmonary artery. MPA - main pulmonary artery, RPA - right pulmonary artery

**Figure 2:**
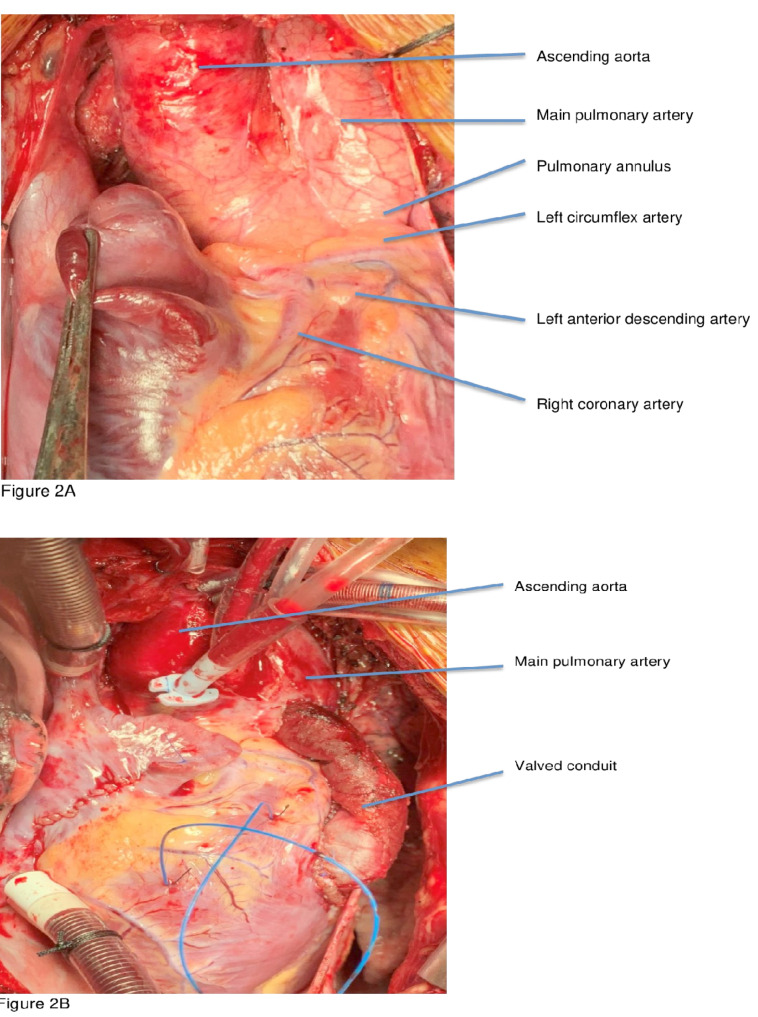
**(A)** Intraoperative view showing a single coronary artery arising from right aortic sinus dividing immediately into right coronary artery, left anterior descending artery and left circumflex artery. Left circumflex artery was seen crossing right ventricular outflow tract close to the pulmonary annulus (**B)** Intraoperative view showing pericardial autologus conduit with reconstructed bicuspid pulmonary valve using polytetrafluoroethylene membrane

**Figure 3:**
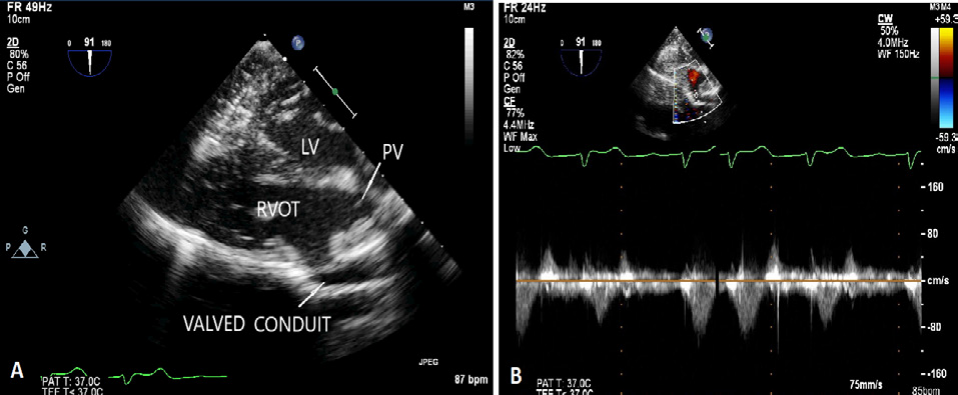
**(A)** Postoperative transesophageal echocardiography showing native unicuspid pulmonary valve and valved conduit. LV - left ventricle, PV - native unicuspid pulmonary valve, RVOT - right ventricular outflow tract (**B)** Postoperative transesophageal echocardiography with pulse wave doppler across the valved pericardial autologous conduit showing minimal gradient of 8 mm Hg

A 11-year-old girl, weighing 21 kg presented with the complaints of dyspnea and cyanosis while playing with room air oxygen saturation of 75-85%. The diagnosis of TOF was confirmed by transesophageal echocardiography and cardiac catheterization study. The cardiac catheterization study showed single coronary artery arising from right aortic sinus with anomalous course of left circumflex artery crossing the RVOT (Figure 1A/Supplementary Video S1). There was absence of the intrapericardial and hilar segments of left pulmonary artery (Figure 1B). Multiple small aortopulmonary collaterals were seen separately supplying the left lung without reformation of the hilar branch pulmonary artery. The right pulmonary artery (Nakata Index Z score +2) and left ventricle (left ventricular end diastolic volume index > 30 ml/m^2^) were normal sized (Figure 1B). Patient was planned for primary single lung intracardiac repair. Intraoperatively, a single coronary artery was arising from right aortic sinus which was immediately dividing into right coronary artery, left anterior descending artery and left circumflex artery (Figure 2A). The right coronary artery had normal course. The left anterior descending artery was crossing RVOT far off from the pulmonary annulus. The left circumflex artery was crossing RVOT, very close to the pulmonary annulus (Figure 2A). Pulmonary annulus was hypoplastic with poststenotic dilatation of main pulmonary artery. The main pulmonary artery was continuing as right pulmonary artery with completely absent left pulmonary artery. The left lung was small and fibrotic with multiple small collaterals seen around the hilum without hilar reformation of the branch pulmonary artery. The handmade valved pericardial autologous conduit (12 mm diameter) with bicuspid pulmonary valve reconstruction using polytetrafluoroethylene membrane was prepared by the technique described by Schlichter et al.^4^ The ventricular septal defect was closed using autologous glutaraldehyde treated pericardium. The hypertrophied infundibular muscle was excised. The native pulmonary valve was unicuspid and hypoplastic. Leaflet tethering was released. Hegar’s dilator of 7 mm size was negotiated through the hypoplastic pulmonary annulus. The valved pericardial autologus conduit was then anastomosed, first at the pulmonary end followed by the ventriculotomy end (Figure 2B). The post-operative right ventricular systolic pressure was 50% of systemic systolic pressure. The transesophageal echocardiography showed gradient of 8 mm Hg across the valved conduit and mild neopulmonary valve regurgitation (Figure 3A, 3B). The patient recovered uneventfully. The patient was discharged on 11^th^ post-operative day and is doing well in the follow-up period.

## Discussion

Single origin coronary artery with ACA crossing the RVOT in TOF has been described in few studies.^5,6,7,8^ The branches of single coronary artery arising from either the facing or non-facing sinus can take various courses after take-off.^5,6,7,8^ The prepulmonic course of left circumflex artery crossing RVOT in TOF with absent left pulmonary artery has not been reported in the literature.

A major coronary branch crossing the RVOT poses a great surgical challenge to the surgeon leading to change the standard way of TOF repair. Injury to this coronary branch can lead to fatal life threatening complications.^1^ Various techniques of RVOT reconstruction in this group of patients include oblique ventriculotomy parallel to the course of ACA, tailored ventriculotomy, two patch repair, transatrial approach, double barrel repair and extracardiac right ventricle-to-pulmonary artery conduit.^7,9^ Despite of proper preparation, damage can still occur to the major anomalous coronary branch crossing RVOT and in that case emergency bypass grafting to the distal end of the severed artery has to be done to restore the blood circulation.^10^

## Conclusion

In conclusion, coronary anatomy should be clearly defined before surgery in patients with anomalous coronary artery crossing the right ventricular outflow tract in tetralogy of Fallot otherwise as it may test the surgeon’s skill and may also need simultaneous use of multiple tools from the surgeon’s armamentarium for successful surgical outcome in these patients.

## Competing interest

None declared.

## Ethical approval

Written informed consent was obtained from the patient for the publication of this case report.

## Funding

No funds were received for this work.

## Supplementary materials

Supplementary file 1 contains Video S1.
